# Mass Drug Administration: Contextual Factor Considerations

**DOI:** 10.4269/ajtmh.22-0767

**Published:** 2024-01-23

**Authors:** Zachary D. Schneider, Alexandra L. Busbee, Marisa C. Boily, Monica P. Shah, Jimee Hwang, Kim A. Lindblade, Julie R. Gutman

**Affiliations:** ^1^ Malaria Branch, Division of Parasitic Diseases and Malaria, Centers for Disease Control and Prevention, Atlanta, Georgia;; ^2^ Rollins School of Public Health, Emory University, Atlanta, Georgia;; ^3^ U.S. President’s Malaria Initiative, Malaria Branch, Division of Parasitic Diseases and Malaria, Centers for Disease Control and Prevention, Atlanta, Georgia;; ^4^ Global Malaria Programme, World Health Organization, Geneva, Switzerland

## Abstract

In designing mass drug administration (MDA) campaigns, it is imperative to consider contextual factors that affect uptake of the intervention, including acceptability, cost, feasibility, and health system considerations, to ensure optimal coverage. We reviewed the literature on contextual factors influencing MDA delivery to provide programs with information to design a successful campaign. From 1,044 articles screened, 37 included contextual factors relevant to participants’ values and preferences, drivers of MDA acceptability, health equity concerns, financial and economic aspects, and feasibility barriers; 13 included relevant modeling data. Key findings were abstracted by two reviewers and summarized. No studies directly assessed values or direct health equity concerns with respect to MDA, which represents an evidence gap as unequal distributions of effects and factors that impact participant acceptability and program feasibility must be considered to ensure equitable access. Participant acceptability was the most widely surveyed factor, appearing in 28 of 37 studies; perceived adverse events were a frequently noted cause of nonparticipation, mentioned in 15 studies. Feasibility considerations included when, where, and how drugs will be delivered and how to address pregnant women, as these can all have substantial implications for participation. Mass drug administration costs (∼$1.04 to $19.40 per person per round) are driven primarily by drug prices, but the delivery mechanism can have varying costs as well, and integration with other interventions may provide cost savings. Both programmatic goals and sociopolitical and economic contexts must be carefully considered before embarking on an MDA program to ensure programmatic success.

## INTRODUCTION

Mass drug administration (MDA) consists of synchronous administration of antimalarial treatment, irrespective of symptoms, to every person living in a defined geographical area (except to those for whom the medicine is contraindicated) and often over repeated rounds.^1^ Mass drug administration works by clearing existing parasites from the target population and preventing new infections as long as the drug remains at a sufficient level in the person’s blood. A number of factors shape the effectiveness of MDA, most notably the coverage, which is affected by acceptability, feasibility, and cost considerations.^2^^–^^4^

Mass drug administration has the potential to reduce community-level transmission through reduction in the human reservoir of infection and prevention of future infections.^3^ Older studies have found large reductions in parasite prevalence immediately after MDA rounds; however, these gains generally were not sustained in the longer term.^5^^,^^6^ With the availability of longer-acting antimalarials and drugs with gametocytocidal effects, there is renewed interest in MDA as an accelerator strategy for malaria elimination.^7^ Given the cost and difficulty in conducting large-scale trials to assess the effectiveness of MDA and the large number of variables to be considered, mathematical modeling studies can provide useful insights to assist countries in designing and optimizing their programs.^3^^,^^8^^–^^13^ In addition, consideration of contextual factors (e.g., values and preferences, acceptability, health equity, financial and economic considerations, feasibility, and health system considerations) is critical to ensure that potential barriers to implementation are recognized and mitigated against in planning implementation to ensure high coverage that optimizes the efficacy of MDA. Failure to adequately sensitize the population in advance of an MDA, address rumors that may arise around the purpose, or take into account people’s preferences can have deleterious effects on acceptability and subsequently on coverage, reducing the effectiveness of a campaign.^14^ Similarly, ensuring that the delivery modality is feasible, convenient, and cost-effective could impact the long-term success of a program. This systematic review of MDA contextual factor data was conducted to inform the process of MDA campaign guideline development at the WHO and subsequent country decision-making.

## MATERIALS AND METHODS

The methods have been described extensively elsewhere^15^^,^^16^ and in the prospectively published protocol [International Prospective Register of Systematic Reviews registration: CRD42021240921]. Contextual factors related to MDA were considered regardless of whether other, more stringent study design criteria were met (e.g., balance of baseline interventions or type of control group).

### Data collection and analysis.

A single search and review process was conducted for both quantitative data related to MDA (e.g., impact of MDA on the incidence of parasitemia and parasite prevalence measured at the population level; these data are presented separately^15^^,^^16^) and contextual factor data, whereby studies including data on contextual factors but not meeting inclusion criteria for the quantitative review were tagged. Data on contextual factors, including values and preferences; acceptability; health equity, equality, and nondiscrimination; financial and economic considerations; feasibility and health system considerations from tagged studies, were abstracted by two people into a standardized form, compared, and summarized. Discrepancies were resolved by discussion. Insights from mathematical modeling on how variation in operational parameters alter the effectiveness of MDA were summarized for the following parameters when available: timing of rounds with respect to the transmission season, number of rounds, spacing of rounds, number of years for the intervention, coverage, adherence, and antimalarial type, dosage, and schedule.

## RESULTS

**Figure 1.**
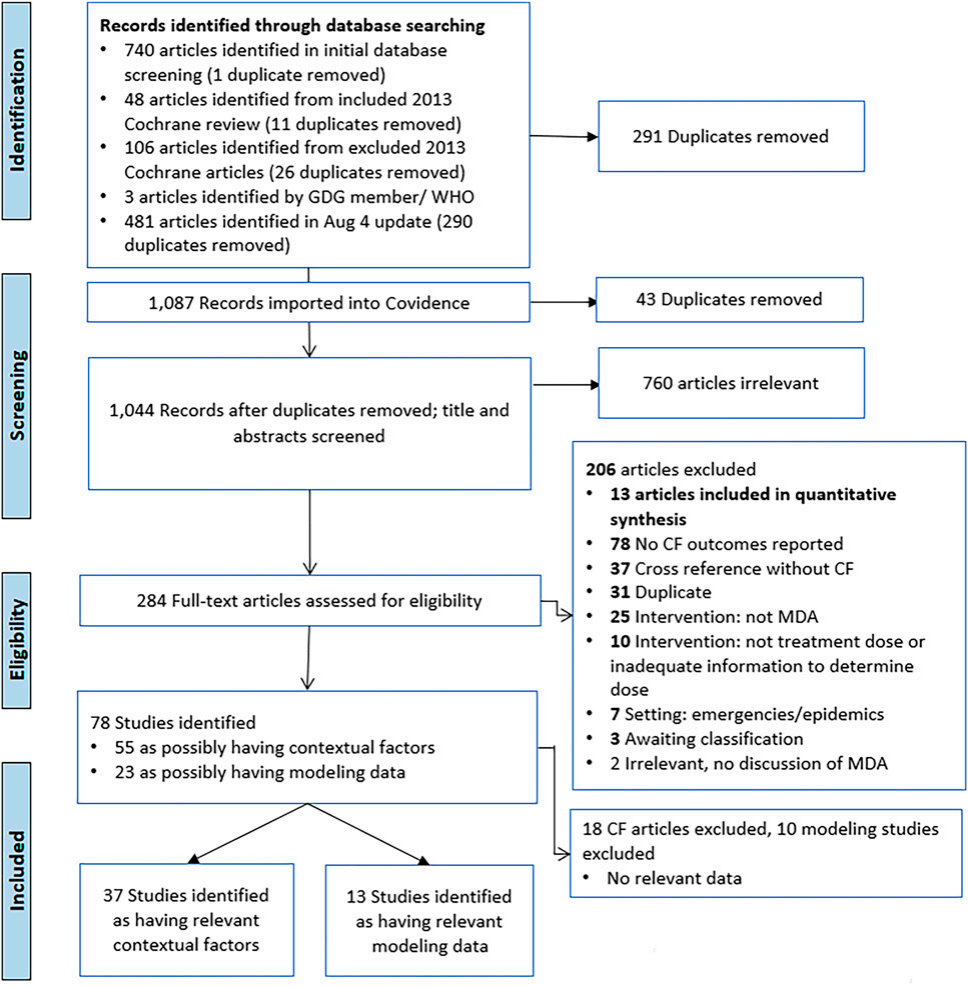
PRISMA flow diagram. CF = contextual factors; GDG = Guideline Development Group; MDA = mass drug administration; PRISMA = Preferred Reporting Items for Systematic Reviews and Meta-Analyses.

A total of 1,378 articles were identified from searching electronic databases, registers, and other sources: 1,221 records from a database search from 2012 onward (date of search: November 11, 2020; updated August 4, 2022), 143 from a previous search conducted for a systematic review on MDA,^5^ and an additional 3 from other sources. After de-duplication, 1,044 articles were screened against title and abstract eligibility. Of these, 55 studies including contextual factors and 23 including modeling were considered; 37 included contextual factors relevant to participants’ values and preferences, drivers of MDA acceptability, health equity concerns, financial and economic aspects of the intervention, and barriers related to feasibility and 13 included relevant modeling data (Figure 1). Efficacy studies of MDA assessed the effects of various combinations of chloroquine, pyrimethamine with or without sulfadoxine/sulfalene, amodiaquine, dihydroartemisinin-piperaquine, primaquine alone or in combination with other agents (most commonly low-dose primaquine), and atebrine plus plasmochine.

### Values and preferences.

Sixteen studies assessed various aspects of values and preferences. One study that surveyed participants’ values found that the desire to protect one’s family or community from future malaria infections was most commonly provided as the rationale for taking MDA.^17^ Participants for whom the concept of healthy people taking antimalarials was novel were favorably disposed toward MDA when they learned that the intervention could eliminate or reduce malaria in their communities.^17^^–^^19^ Reasons for nonparticipation or nonadherence differed between those who did not intend to take part (detailed under the Acceptability section) and those who were unable to take part (detailed under the Feasibility section). Adult participants expressed preferences for interventions that did not cause adverse events (AEs) or did not disrupt economic livelihood^17^^,^^18^^,^^20^^–^^27^; one study in a low-transmission area noted that the “need to earn income may be prioritized above the perceived risk of contracting an otherwise rare disease.”^28^ The perceived effectiveness of MDA determined policymakers’ and authority figures’ support for MDA. One study noted that policymakers were unsupportive because they felt that MDA was not sufficiently supported by scientific evidence, whereas another study found that MDA was widely supported by policymakers because they hoped it would increase the impact of vector control interventions.^14^^,^^29^ The conflicting values between participants and policymakers was noted in one study in which community members stated a preference for MDA during the dry season because it might impact farming in the wet season, whereas scientific and policy figures favored administration of MDA during the wet season because farmers were more likely to travel away from home during the dry season.^22^ Children’s preferences were more difficult to document as they were enrolled by parents, but two studies noted that children’s preferences were based on medication taste and size.^30^^,^^31^

### Acceptability.

Participant acceptability was the most widely surveyed factor, appearing in 28 of 37 studies.^14^^,^^17^^–^^30^^,^^32^^–^^44^ These studies assessed MDA with chloroquine + primaquine or pyrimethamine, dihydroartemisinin-piperaquine (DP) with/without primaquine, primaquine alone, and ivermectin. In most cases of combination therapy with primaquine, low-dose primaquine was used. Overall, acceptability of MDA was high (92%); this was largely driven by concern for one’s personal health and the desire to help the broader community.^28^ In both the Dominican Republic and along the Thai-Burmese border, more affluent individuals were less inclined to participate in MDA; the study authors hypothesized that this might be due to less personal exposure to malaria or to a greater ability to access healthcare if they did become sick.^23^^,^^28^^,^^43^ In Myanmar, factors contributing to willingness to participate in an MDA campaign were “older age [adjusted odds ratio (aOR): 2.38, 95% CI: 1.08–8.96], secondary education level (aOR: 3.99, 95% CI: 1.12–20.01), having good knowledge of malaria (aOR: 2.12, 95% CI: 1.04–4.76), experiencing malaria within the family (aOR: 1.92, 95% CI: 1.14–5.13), and believing that eliminating malaria from the village is possible (aOR: 2.83, 95% CI: 1.07–4.07).”^19^ Local healthcare professionals believed that poor understanding of MDA would limit adherence.^21^ The most common barrier to acceptability, noted by 15 studies (37%), was the fear of perceived AEs or drug–drug interactions with their chronic medications. One respondent refused to participate in MDA owing to the perception that AEs would be worse than malaria.^25^ Conversely, another study noted that participants interpreted mild AEs as evidence of the intervention drug’s effectiveness.^20^ Three studies noted that AEs caused greater demand for local healthcare^18^^,^^26^^,^^27^; concerns about AEs were alleviated by the provision of free healthcare as part of the intervention. Provision of free healthcare generally drove participant engagement and participation^18^^,^^24^^,^^42^; however, in one study, the presence of expatriate physicians and an ambulance heightened concerns about potential side effects.^27^

Two studies found that the concerns about AEs had an economic as well as a health basis.^20^^,^^22^ In both studies, participants cited the proximity in time between harvest season and MDA administration as a reason why AEs could have an undue economic effect. However, respondents in one study used the same logic to reach the opposite conclusion, as they perceived that cases of malaria were more likely than AEs to limit economic activity during harvest season.^29^ Concerns about economic impacts extended to urban populations as well. One study noted that “participating in the campaign requires closing up the shop for nine mornings to come to the healthcare centre” in addition to existing concerns about the lack of personal benefit.^23^ This aspect is closely tied to values/preferences and how people weigh the risk/benefit of malaria versus MDA.

Prior experience with MDA (including MDA for diseases other than malaria) reinforced initial perceptions of MDA. Individuals who had been part of previous MDA trials shared stories among the communities, and if those experiences were poor, community members had negative impressions of MDA.^14^^,^^23^ Reported acceptability of MDA increased from 62% before MDA to 98% after, perhaps in part because the proportion of respondents who answered that MDA could cause side effects decreased from 30% to 20% in the same time frame.^17^

Common themes among analyses of drivers of acceptance were sensitization or education about the intervention, support from a range of local authority figures, and additional health support. From two studies that assessed how participants preferred to learn about MDA, village meetings were the preferred method, followed by radio or television messaging and direct messages from healthcare workers.^32^^,^^42^ In a study in Magude, Mozambique, community members suggested that schools and churches be used to engage with community members, noting the importance of ensuring that sensitization of the population was vital to the success of the program.^37^ One study suggested the need to use social media platforms to better reach younger people.^28^ Adhikari et al.^33^ reported that “Respondents who felt that they have received enough information…were more likely to participate in all rounds of MDA,” a theme that was repeated by five other studies.^22^^,^^23^^,^^25^^,^^35^^,^^40^ Health education was noted as a critical factor to ensure that participants were adherent to the full regimen,^19^ with health professionals noting that asymptomatic people may be unlikely to take a full course of medicine. This was particularly noted with respect to the duration of therapy and large number of pills required when using primaquine for eradication of *Plasmodium vivax* hypnozoites,^19^ as the 14-day course is considerably longer than that of most other antimalarial regimens.^45^

Participants’ education was also reported as a driver linking exposure to health institutions and experience with malaria treatment to increased acceptance of MDA.^42^ However, misinformation, rumors, or previous poor experience with MDA decreased participation, including previous non-malaria MDA efforts such as a prior MDA for lymphatic filariasis that created persistent rumors.^14^^,^^20^^,^^37^ Multiple studies noted that including a range of authority figures, both official and nonofficial, increased acceptance.^23^^,^^25^^,^^43^ This included figures from different domains (e.g., governmental figures, religious leaders, and health authorities) as well as ensuring that authority figures did not exclude certain groups. Ensuring support of armed groups was sometimes necessary, as was ensuring that conflicting groups did not perceive MDA as favoring one side over another.^43^

Efforts to reduce the perception of the intervention staff as outsiders or gain the local populations’ trust were important to ensure successful implementation.^20^^,^^27^^,^^43^ One study noted that areas that had experienced prolonged violence were more likely to demand support from local figures.^44^ Several studies noted accidental cultural conflicts, such as suspicion of a culturally inappropriate informed consent process, or the inadvertent exclusion of certain groups of women through the requirement of pregnancy testing.^20^^,^^27^^,^^43^ In a study in Myanmar, “a small group of villagers said they would refuse to participate should any of the staff be Muslim,” reflecting national tensions.^43^

### Feasibility and health systems challenges.

Overall, 19 studies discussed issues related to feasibility. Twelve studies mentioned MDA administration challenges related to remoteness or mobile populations; this may be particularly true in areas where there are migrant workers.^19^^,^^20^^,^^23^^,^^25^^,^^35^^,^^38^^,^^39^^,^^42^^,^^44^^,^^46^^–^^48^ Of these, three studies noted that absenteeism from home was one of the major driving forces behind nonadherence (and one felt that determining participants’ mobility and seasonal locations prior to MDA contributed to the success of the campaign).^23^^,^^35^^,^^44^^,^^47^ One study in Cambodia highlighted that MDA was logistically easier in the dry season because the majority of agricultural work is conducted in the wet season.^42^ Three studies noted problems related to timing other than seasonal weather and agricultural patterns: overlaps with religious events, especially including fasting,^21^ unpredictable policy changes at the national level,^14^ and the school year.^48^ Feasibility concerns related to participant’s religion were further noted by one study that attempted to implement a program where healthcare workers observed MDA drugs being taken but found that some women were unwilling to remove face coverings in front of strangers.^30^ This was resolved by creating sequestered administration sites staffed by accepted local staff. In addition, one study commented that survey respondents may have felt that nonadherence due to travel was more socially acceptable than nonadherence due to institutional mistrust, and this may have altered survey results.^23^ Delivery modality (fixed clinics versus house-to-house delivery) was explored in one study; compensation for travel time was provided to those who presented to the clinics but not to those who received the drug at home, and coverage was reported to be similar regardless of delivery modality.^42^

Another common pattern related to refusal or nonadherence among otherwise willing participants was the taste, size, and number of pills. Four studies noted participant complaints that the tablets they were given were too bitter (especially chloroquine), too large to swallow, or too numerous to manage.^22^^,^^31^^,^^41^ Of these, two drew explicit contrasts between large and bitter chloroquine pills and smaller tasteless pyrimethamine pills, both noting that the difference was especially pronounced in children, observing that “children vomit or reject chloroquine and other bitter antimalarial drugs, but will accept the tasteless pyrimethamine readily.”^30^^,^^31^ The specific drugs that were assessed in studies reporting on feasibility included chloroquine + primaquine, pyrimethamine, sulfadoxine-pyrimethamine + primaquine, and DP with or without primaquine.

### Health equity, equality, and nondiscrimination.

As noted above, links between knowledge about malaria and MDA acceptability were common. Two studies noted that MDA acceptability was associated with formal education and that educational disparities led to inequities in MDA adherence, but they did not agree on the direction of the association.^23^^,^^43^ Literacy, formal education, and mobility were found to be linked to MDA participation as well.^34^

One study required women of reproductive age to undergo pregnancy testing; both the production of the urine specimen for testing in places where a private toilet was not available and reading of the test result in a public place created privacy concerns, leading to decreased participation among this group.^36^ Ethnicity was related to willingness to participate in MDA.^40^ One study noted that ethnic minorities were potentially underrepresented because of geographic distribution rather than direct discrimination and also found that some portions of the population relied on traditional medicine because of affordability more so than any other reason, which led to less familiarity with MDA and thus to lower participation.^42^^,^^43^ Exposure to and access to health facilities were associated with improved adherence to MDA, and studies that provided access to primary care noted this improved adherence, especially in populations who had previously lacked access to healthcare.^18^^,^^43^^,^^44^

### Financial and economic factors.

The cost of MDA varied from ∼$1.04 to $19.40 USD per person per round; one study estimated that drugs accounted for 70% of the cost of MDA.^49^ On the lower end, Gabaldon and Guerrero^50^ estimated a cost of $0.11 to $0.21 USD per person per visit, depending on distance between houses, (approximately $1.04 to $1.99 in 2021 USD). Cirera et al.^51^ reported substantially higher costs, with an average yearly cost of $20.70 USD per targeted person — $26 USD for rounds 1 and 2 to $13 USD for rounds 3 and 4 per person treated per round. Galactionova et al.^49^ estimated an initial cost of $2.35 USD per person treated per round in the first year, dropping to $2.19 USD per person per round if implemented annually for 5 years, and that MDA would be more expensive than other interventions such as rapid reporting, reactive case detection, or indoor residual spraying. This same study also noted that aspects such as sensitization and training may overlap between programs such as MDA and reactive case detection and that co-implementation might provide overall improvements in cost-effectiveness.^49^

Yukich et al.^52^ estimated that the costs of MDA and focal MDA per person targeted and reached were similar, with a cost of approximately $4.71 per person reached per round but that “MDA was superior in all cost-effectiveness measures, including cost per infection averted, cost per case averted, cost per death averted, and cost per disability-adjusted life year averted.” Furthermore, the cost of MDA per person reached was substantially lower ($2.90 USD) in an operational setting. It was also noted that “[MDA compared to focal MDA] showed superior cost-effectiveness in terms of infections averted and appeared to improve when used in relatively higher infection incidence settings.”^52^

### Mathematical modeling.

#### Timing, number, and/or spacing of rounds and number of years of the intervention.

Consistent with trial data, models predicted that a single round of MDA would lead to an initial decrease in infections but that the duration of effect would be short-lived. Application of additional rounds is predicted to substantially improve the impact and duration of effect. Gerardin et al.^53^ found that three rounds had a greater impact on parasite prevalence than two, noting that with DP “[at] 70% coverage, prevalence one-month post-campaign is more than twice as high for a 2-round campaign as a 3-round campaign….” Walker et al.^54^ found that with 90% coverage at every round, each additional round of MDA increased the proportion of the population in whom pre-elimination was achievable (74.9% [95% CI: 72.3–81.3%] with one round of treatment per year, 81.6% [81.3–88.4%] with two rounds per year, and 91.4% [85.2–93.2%] with three rounds per year). Brady et al.^3^ noted that the effects of three versus two rounds were primarily a result of reaching additional individuals in the third round who had not received treatment in prior rounds, and 2 years of interventions were superior to a single year. Silal et al.^55^ found that six consecutive 2-monthly rounds of MDA substantially decreased infections and that it took approximately 2 years to recover to pre-MDA levels after the end of MDA. Even in areas with low to very low malaria prevalence at baseline, it is estimated that elimination will require multiple rounds per year for a number of years.^56^^,^^57^

Mass drug administration achieves highest coverage when distributed during times when people are not traveling away from their homes (e.g., for religious or economic/work reasons) and during the low-transmission season; correct timing with respect to travel was more critical than transmission season.^3^^,^^58^ On the other hand, Silal et al.^55^ noted that application in peak transmission season resulted in an approximately 20% greater decline in infections than application in the trough season. Maude et al.^59^ reported that the maximal impact of MDA was noted if the final round of MDA was completed before the nadir of seasonal parasite prevalence; three rounds of MDA with an artemisinin-based combination therapy (ACT) over 3 months was found to be optimal. The second round of MDA should be given as soon as possible after the first (i.e., within 1–2 months of commencing the first round).^60^ No substantial difference was noted between rounds spaced 4 versus 6 weeks apart.^3^

#### Coverage.

Mass drug administration effectiveness relies on high intervention coverage, that is, when a large proportion of the entire population receives one or more rounds of MDA.^3^^,^^59^ Coverage is the most important operational factor determining effectiveness, and in the case of multiple MDA rounds, MDA is less effective if the same proportion of the population is consistently missed by treatment rounds.^3^ High coverage may be difficult to achieve as a result of people who are excluded because of contraindications to the antimalarial, refusals, and absence during treatment rounds. Although conducting repeated rounds provides an opportunity to improve coverage, the same people may be repeatedly excluded, reducing effective coverage. Mass drug administration effectiveness is nonlinearly related to intervention coverage; efficacy improves more rapidly with improvements in coverage at the lower end of coverage levels than at the higher end.^60^ An increase in effective coverage from 30% to 70% resulted in a substantial increase in predicted median reduction in the *Plasmodium falciparum* parasite rate measured by polymerase chain reaction, from 15% (range across models: 10–30%) to 61% (range: 19–64%).^3^ Although coverage may be greatly influenced by the modality of MDA delivery (fixed post versus house-to-house delivery), none of the models included this factor, focusing on achieved coverage.

Modeling from Maude et al.^59^ suggests that although use of MDA would accelerate the process of elimination, if treatment with an ACT for acute episodes of malaria was “continued for long enough at high coverage, this alone [would] be sufficient” for elimination, as long as there was a substantial enough proportion of immune people who developed symptoms. The duration required to achieve elimination varies widely (from 1.2 to 23.2 years) depending on the duration of immunity and what proportion of the immune population remains entirely asymptomatic.^59^ Maude et al.^59^ also noted that MDA would be most impactful delivered as three rounds over 3 months, starting as infection prevalence is dropping, with the last dose administered before the seasonal nadir in parasite prevalence. Pemberton-Ross et al.^61^ suggest that MDA is unlikely to achieve elimination in areas with a large population (>1,000) or high reproductive number under control ([Rc] >1.2). Specifically, this model suggests that “[e]limination on operationally relevant timelines (<10 years) at 90% MDA coverage is not expected in populations >200 unless Rc ≪1.1,”^61^ suggesting that MDA is most efficient as a strategy in areas of very low transmission.

Modeling the combination of strategies that would maximize impact and minimize cost to achieve a national prevalence of 1% in mainland Tanzania, Runge et al.^62^ suggested that MDA coverage of 80% in most areas in conjunction with other interventions (insecticide-treated nets, indoor residual spraying, and improved case management) is needed.

#### Dosage and dosage schedule.

Although Gerardin et al.^53^ noted a difference in efficacy with DP versus artemether-lumefantrine (AL), Stuckey et al.^56^ found no effect on parasite prevalence of changing drugs from DP to AL. In addition, Stuckey et al.^56^ found no impact from the addition of single low-dose primaquine or ivermectin to DP. In contrast, other models by Slater et al.^63^ found that the addition of ivermectin would result in a greater reduction in rapid diagnostic test (RDT) positivity and a more sustained period of reduced RDT positivity than MDA with AL alone, although Maude et al.^59^ found a beneficial effect of adding primaquine to ACT. Robinson et al.^64^ noted that the addition of an 8-aminoquinoline to a blood stage drug is required for a sustained reduction in *P. vivax* prevalence; tafenoquine is estimated to be more effective than primaquine owing to a longer duration of causal prophylaxis. Compared with the use of ACTs, resistance to atovaquone proguanil develops quickly, with the predicted loss of atovaquone proguanil efficacy as a treatment within 4 years, and is not recommended for treatment of acute infection or MDA.^60^

#### Travel/human mobility.

Mass drug administration is predicted to be most successful when conducted during seasons when people are not traveling and least successful when people are traveling, particularly when this occurs during peak malaria seasons in areas with higher malaria risk.^58^

## DISCUSSION

For MDA to be successful, high coverage of the target population must be achieved. Although MDA is generally acceptable, especially with good understanding of the intervention, the occurrence of AEs or the perception that they will occur impacts acceptability. This needs to be recognized and considered in the choice of regimens. In addition, the timing and potential economic impact of any campaign must be considered to ensure high coverage. Ensuring that a community is well sensitized and understands the rationale for the MDA and that the messaging is delivered through trusted channels is critical to preventing rumors that can negatively impact participation. Although no studies directly assessed values with respect to MDA, all studies that surveyed participants found that respondents did value reducing malaria cases but that different groups valued different aspects of MDA administration. Likewise, no studies reported direct health equity concerns. However, unequal distributions of effects and factors that impact participant acceptability and program feasibility have the potential to present a major concern. Participant acceptability was the most widely surveyed factor; perceived AEs were the biggest cause of nonparticipation. Increasing awareness of the intervention and education about malaria were universally recognized as critical to ensuring participation and adherence. Involving a range of local authorities was essential. Physical distance and mobility, especially seasonal mobility, were major drivers of nonadherence among populations who were otherwise willing to participate. The taste, size, and number of pills for MDA also contributed to on-the-spot refusal or nonadherence, especially among children. Feasibility considerations to be thought through include where and how drugs will be delivered and what will be done in the case of pregnant women as well as women of reproductive age, as this can have substantial implications for participation. Costs of the implementation are driven primarily by drug prices, but the delivery mechanism can have varying costs as well. The studies that assessed financial considerations produced a wide range of costs per patient but suggested that cost-effectiveness may be improved by taking advantage of overlaps between programs and that cost-effectiveness may vary in settings based on transmission intensity. Costs must be carefully considered in the context of program goals, as evidence from China suggests that a sustained MDA program, delivered in the context of a package of interventions, is likely to have much greater impact than a program that provides only a few rounds or that is conducted without maximizing other intervention coverage^12^^,^^65^^,^^66^; this is further supported by results from mathematical modeling.^3^ The sociopolitical and economic context is critical to determine the relative success and impact of the program, and the importance of careful planning for an MDA campaign with a good understanding of the various contextual factors in the specific location cannot be understated.^23^

## References

[1] World Health Organization , 2021. *WHO Malaria Terminology*. Geneva, Switzerland: WHO. Available at: https://www.who.int/publications/i/item/9789240038400. Accessed October 2, 2023.

[2] Bousema T Drakeley C, 2011. Epidemiology and infectivity of *Plasmodium falciparum* and *Plasmodium vivax* gametocytes in relation to malaria control and elimination. Clin Microbiol Rev 24: 377–410. 21482730 10.1128/CMR.00051-10 PMC3122489

[3] Brady OJ , 2017. Role of mass drug administration in elimination of *Plasmodium falciparum* malaria: a consensus modelling study. Lancet Glob Health 5: e680–e687. 28566213 10.1016/S2214-109X(17)30220-6 PMC5469936

[4] Okell L Pemberton-Ross P Wenger E, 2015. *Consensus Modelling Evidence to Support the Design of Mass Drug Administration Programmes*. Malaria Policy Advisory Committee Meeting, September 16-18, 2015, Geneva, Switzerland.

[5] Poirot E Skarbinski J Sinclair D Kachur SP Slutsker L Hwang J, 2013. Mass drug administration for malaria. Cochrane Database Syst Rev 12: CD008846. 10.1002/14651858.CD008846.pub2 PMC4468927 24318836

[6] Shah MP Hwang J Choi L Lindblade KA Kachur SP Desai M, 2021. Mass drug administration for malaria. Cochrane Database Syst Rev 9: CD008846. 10.1002/14651858.CD008846.pub3 PMC8479726 34585740

[7] Eisele TP, 2019. Mass drug administration can be a valuable addition to the malaria elimination toolbox. Malar J 18: 281. 31438950 10.1186/s12936-019-2906-8 PMC6704699

[8] Okell LC Griffin JT Kleinschmidt I Hollingsworth TD Churcher TS White MJ Bousema T Drakeley CJ Ghani AC, 2011. The potential contribution of mass treatment to the control of *Plasmodium falciparum* malaria. PLoS One 6: e20179. 21629651 10.1371/journal.pone.0020179 PMC3101232

[9] Kaneko A Taleo G Kalkoa M Yamar S Kobayakawa T Bjorkman A, 2000. Malaria eradication on islands. Lancet 356: 1560–1564. 11075770 10.1016/S0140-6736(00)03127-5

[10] Chen WI, 1991. Malaria eradication in Taiwan, 1952–1964 – some memorable facts. Gaoxiong Yi Xue Ke Xue Za Zhi 7: 263–270. 2056560

[11] Taiwan Provincial Malaria Research Institute, WHO Malaria Team in Taiwan , 1958. Malaria control and eradication in Taiwan: progress report, May 1952 50 June 1957. Bull World Health Organ 19: 595–620. 13596886 PMC2537726

[12] Li X-H Zhou H-N Xu J-W Lin Z-R Sun X-D Li J-Y Lin X-X Xie Y Alonso P Yang H-L, 2021. Seven decades towards malaria elimination in Yunnan, China. Malar J 20: 147. 33711990 10.1186/s12936-021-03672-8 PMC7953382

[13] Xu X Wang JJ Jiang JJ Zhang T Lv XF Wang SQ Liu ZJ Li WD Lu XC, 2022. Mass drug administration in response to vivax malaria resurgence in Anhui Province of Huanghuai Plain, China. Adv Parasitol 116: 115–152. 35752446 10.1016/bs.apar.2022.04.001

[14] Kaehler N Adhikari B Cheah PY Day NPJ Paris DH Tanner M Pell C, 2019. The promise, problems and pitfalls of mass drug administration for malaria elimination: a qualitative study with scientists and policymakers. Int Health 11: 166–176. 30395228 10.1093/inthealth/ihy079 PMC6484636

[15] Tussell M , 2024. Development of systematic reviews to inform WHO's recommendations for elimination and prevention of re-establishment of malaria: Methodology. Am J Trop Med Hyg 110 * (* *Suppl 4* *): * 11–16. 38118164 10.4269/ajtmh.22-0740 PMC10993789

[16] Schneider ZD Shah MP Boily MC Busbee AL Hwang J Lindblade KA Gutman JR, 2024. Mass drug administration to reduce malaria transmission: A systematic review and meta-analysis. Am J Trop Med Hyg 110 * (* *Suppl 4* *): * 17–29. 38118174 10.4269/ajtmh.22-0766 PMC10993786

[17] Silumbe K , 2020. Assessment of the acceptability of testing and treatment during a mass drug administration trial for malaria in Zambia using mixed methods. Am J Trop Med Hyg 103: 28–36. 10.4269/ajtmh.19-0663 PMC7416978 32618242

[18] Adhikari B , 2018. Why do people participate in mass anti-malarial administration? Findings from a qualitative study in Nong District, Savannakhet Province, Lao PDR (Laos). Malar J 17: 15. 29316932 10.1186/s12936-017-2158-4 PMC5761145

[19] Aung PL , 2021. The acceptability of targeted mass treatment with primaquine for local elimination of vivax malaria in a northern Myanmar township: a mixed-methods study. Parasit Vectors 14: 549. 34689796 10.1186/s13071-021-05064-y PMC8543804

[20] Dierickx S Gryseels C Mwesigwa J O’Neill S Bannister-Tyrell M Ronse M Jaiteh F Gerrets R D’Alessandro U Grietens KP, 2016. Factors associated with non-participation and non-adherence in directly observed mass drug administration for malaria in The Gambia. PLoS One 11: e0148627. 26866685 10.1371/journal.pone.0148627 PMC4750858

[21] Ali AS , 2017. Artemisinin combination therapy mass drug administration in a setting of low malaria endemicity: programmatic coverage and adherence during an observational study in Zanzibar. Malar J 16: 332. 28807035 10.1186/s12936-017-1982-x PMC5557321

[22] Dial NJ Ceesay SJ Gosling RD D’Alessandro U Baltzell KA, 2014. A qualitative study to assess community barriers to malaria mass drug administration trials in The Gambia. Malar J 13: 47. 24495715 10.1186/1475-2875-13-47 PMC3915615

[23] Kajeechiwa L , 2016. The acceptability of mass administrations of anti-malarial drugs as part of targeted malaria elimination in villages along the Thai-Myanmar border. Malar J 15: 494. 27677694 10.1186/s12936-016-1528-7 PMC5039796

[24] Murta FLG Mendes MO Sampaio VS Junior ASB Diaz-Bermudez XP Monteiro WM Lacerda MVG, 2019. Misperceptions of patients and health workers regarding malaria elimination in the Brazilian Amazon: a qualitative study. Malar J 18: 223. 31272509 10.1186/s12936-019-2854-3 PMC6611038

[25] Pell C , 2017. Mass anti-malarial administration in western Cambodia: a qualitative study of factors affecting coverage. Malar J 16: 206. 28526019 10.1186/s12936-017-1854-4 PMC5438518

[26] Pell CL , 2019. Community engagement, social context and coverage of mass anti-malarial administration: comparative findings from multi-site research in the Greater Mekong sub-region. PLoS One 14: e0214280. 30908523 10.1371/journal.pone.0214280 PMC6433231

[27] Peto TJ , 2018. Community participation during two mass anti-malarial administrations in Cambodia: lessons from a joint workshop. Malar J 17: 53. 29374462 10.1186/s12936-018-2202-z PMC5787251

[28] Keys H Ureña K Reyes J Bardosh K Pell C Puello J Blount S Noland GS, 2021. Rapid ethnographic assessment for potential anti-malarial mass drug administration in an outbreak area of Santo Domingo, Dominican Republic. Malar J 20: 76. 33557830 10.1186/s12936-021-03594-5 PMC7869078

[29] Wanzira H Naiga S Mulebeke R Bukenya F Nabukenya M Omoding O Echodu D Adoke Y, 2018. Community facilitators and barriers to a successful implementation of mass drug administration and indoor residual spraying for malaria prevention in Uganda. Am J Trop Med Hyg 99 *(* *Suppl* *):* 349. 10.1186/s12936-018-2624-7 PMC6298012 30558632

[30] Archibald HM, World Health Organization, 1960. Field Trials of Mass Administration of Antimalarial Drugs in Northern Nigeria/by H. M. Archibald. Available at: https://iris.who.int/handle/10665/64729; Accessed August 25, 2022.

[31] Jones SA, 1958. Mass treatment with pyrimethamine; a study of resistance and cross resistance resulting from a field trial in the hyperendemic malarious area of Makueni, Kenya. September 1952–September 1953. Trans R Soc Trop Med Hyg 52: 547–561. 13625373 10.1016/0035-9203(58)90104-4

[32] Adhikari B Pell C Phommasone K Soundala X Kommarasy P Pongvongsa T Henriques G Day NPJ Mayxay M Cheah PY, 2017. Elements of effective community engagement: lessons from a targeted malaria elimination study in Lao PDR (Laos). Glob Health Action 10: 1366136. 28914184 10.1080/16549716.2017.1366136 PMC5645700

[33] Adhikari B , 2017. Factors associated with population coverage of targeted malaria elimination (TME) in southern Savannakhet Province, Lao PDR. Malar J 16: 424. 29061133 10.1186/s12936-017-2070-y PMC5653989

[34] Adhikari B , 2018. Perceptions of asymptomatic malaria infection and their implications for malaria control and elimination in Laos. PLoS One 13: e0208912. 30533024 10.1371/journal.pone.0208912 PMC6289463

[35] Chiyende E Silumbe K Sikombe C Wilhelm E Jennings T Miller JM, 2016. Targeted community sensitization to reduce anticipated refusals in malaria mass drug administration trial: lessons learned from southern Zambia. Am J Trop Med Hyg 95 *(* *Suppl 1* *):* 485.

[36] Fehr A , 2021. From informed consent to adherence: factors influencing involvement in mass drug administration with ivermectin for malaria elimination in The Gambia. Malar J 20: 198. 33902611 10.1186/s12936-021-03732-z PMC8073909

[37] Galatas B , 2021. Community acceptability to antimalarial mass drug administrations in Magude district, southern Mozambique: a mixed methods study. PLoS One 16: e0249080. 33755685 10.1371/journal.pone.0249080 PMC7987150

[38] Kajeechiwa L Thwin MM Nosten S Tun SW Parker D von Seidlein L Tangseefa D Nosten F Cheah PY, 2017. Community engagement for the rapid elimination of malaria: the case of Kayin State, Myanmar. Wellcome Open Res 2: 59. 28894847 10.12688/wellcomeopenres.12051.1 PMC5580421

[39] Kaneko A, 2010. A community-directed strategy for sustainable malaria elimination on islands: short-term MDA integrated with ITNs and robust surveillance. Acta Trop 114: 177–183. 20132788 10.1016/j.actatropica.2010.01.012

[40] Nguyen T-N , 2017. Community perceptions of targeted anti-malarial mass drug administrations in two provinces in Vietnam: a quantitative survey. Malar J 16: 17. 28061908 10.1186/s12936-016-1662-2 PMC5216593

[41] Ossi GT, 1967. An epidemic in the life of a malaria eradication programme. Bull Endem Dis (Baghdad) 9: 5–18.

[42] Peto TJ , 2018. The feasibility and acceptability of mass drug administration for malaria in Cambodia: a mixed-methods study. Trans R Soc Trop Med Hyg 112: 264–271. 29917147 10.1093/trstmh/try053 PMC6044409

[43] Sahan K , 2017. Community engagement and the social context of targeted malaria treatment: a qualitative study in Kayin (Karen) State, Myanmar. Malar J 16: 75. 28196536 10.1186/s12936-017-1718-y PMC5310060

[44] Tangseefa D , 2018. “Nine Dimensions”: a multidisciplinary approach for community engagement in a complex postwar border region as part of the targeted malaria elimination in Karen/Kayin State, Myanmar. Wellcome Open Res 3: 116. 30687790 10.12688/wellcomeopenres.14698.1 PMC6343222

[45] Shah MPWN Lindblade KA Hwang J, 2023. Mass relapse prevention to reduce transmission of *Plasmodium vivax* – A systematic review. Am J Trop Med Hyg 110 * (* *Suppl 4* *): * 38–43. 38118171 10.4269/ajtmh.22-0727 PMC10993785

[46] Bever CA Gerardin J Hamainza B Conner R Miller J Eckhoff PA Earle D Wenger EA, 2016. Epidemiological and operational lessons learned from a malaria elimination campaign in Zambia’s Lake Kariba region. Am J Trop Med Hyg 95 *(* *Suppl 1* *):* 490.30851018

[47] Chang MA , 2019. Results of a pilot of targeted mass drug administration with sulfadoxine-pyrimethamine and primaquine as a component of a malaria elimination package in Haiti. Am J Trop Med Hyg 101 *(* *Suppl* *):* 417.

[48] Mulebeke R Wanzira H Bukenya F Eganyu T Collborn K Elliot R Van Geertruyden J-P Echodu D Yeka A, 2019. Implementing population-based mass drug administration for malaria: experience from a high transmission setting in north eastern Uganda. Malar J 18: 271. 31399051 10.1186/s12936-019-2902-z PMC6688214

[49] Galactionova K Velarde M Silumbe K Miller J McDonnell A Aguas R Smith TA Penny MA, 2020. Costing malaria interventions from pilots to elimination programmes. Malar J 19: 332. 32928227 10.1186/s12936-020-03405-3 PMC7491157

[50] Gabaldon A Guerrero L, 1959. An attempt to eradicate malaria by the weekly administration of pyrimethamine in areas of out-of-doors transmission in Venezuela. Am J Trop Med Hyg 8: 433–439. 13670370 10.4269/ajtmh.1959.8.433

[51] Cirera L , 2020. Moving towards malaria elimination in southern Mozambique: cost and cost-effectiveness of mass drug administration combined with intensified malaria control. PLoS One 15: e0235631. 32628741 10.1371/journal.pone.0235631 PMC7337313

[52] Yukich JO , 2020. Cost-effectiveness of focal mass drug administration and mass drug administration with dihydroartemisinin-piperaquine for malaria prevention in Southern Province, Zambia: results of a community-randomized controlled trial. Am J Trop Med Hyg 103: 46–53. 32618249 10.4269/ajtmh.19-0661 PMC7416981

[53] Gerardin J Eckhoff P Wenger EA, 2015. Mass campaigns with antimalarial drugs: a modelling comparison of artemether-lumefantrine and DHA-piperaquine with and without primaquine as tools for malaria control and elimination. BMC Infect Dis 15: 144. 25887935 10.1186/s12879-015-0887-y PMC4376519

[54] Walker PGT Griffin JT Ferguson NM Ghani AC, 2016. Estimating the most efficient allocation of interventions to achieve reductions in *Plasmodium falciparum* malaria burden and transmission in Africa: a modelling study. Lancet Glob Health 4: e474–e484. 27269393 10.1016/S2214-109X(16)30073-0

[55] Silal SP Little F Barnes KI White LJ, 2014. Towards malaria elimination in Mpumalanga, South Africa: a metapopulation modeling approach. Malar J 13: 297. 25086861 10.1186/1475-2875-13-297 PMC4127654

[56] Stuckey EM Miller JM Littrell M Chitnis N Steketee R, 2016. Operational strategies of anti-malarial drug campaigns for malaria elimination in Zambia’s Southern Province: a simulation study. Malar J 15: 148. 26957364 10.1186/s12936-016-1202-0 PMC4784285

[57] Bretscher MT Griffin JT Ghani AC Okell LC, 2017. Modelling the benefits of long-acting or transmission-blocking drugs for reducing *Plasmodium falciparum* transmission by case management or by mass treatment. Malar J 16: 341. 28814310 10.1186/s12936-017-1988-4 PMC5559805

[58] Gerardin J Bertozzi-Villa A Eckhoff PA Wenger EA, 2018. Impact of mass drug administration campaigns depends on interaction with seasonal human movement. Int Health 10: 252–257. 29635471 10.1093/inthealth/ihy025 PMC6031018

[59] Maude RJ , 2012. Optimising strategies for *Plasmodium falciparum* malaria elimination in Cambodia: primaquine, mass drug administration and artemisinin resistance. PLoS One 7: e37166. 22662135 10.1371/journal.pone.0037166 PMC3360685

[60] Maude RJ Nguon C Dondorp AM White LJ White NJ, 2014. The diminishing returns of atovaquone-proguanil for elimination of *Plasmodium falciparum* malaria: modelling mass drug administration and treatment. Malar J 13: 380. 25249272 10.1186/1475-2875-13-380 PMC4192368

[61] Pemberton-Ross P Chitnis N Pothin E Smith TA, 2017. A stochastic model for the probability of malaria extinction by mass drug administration. Malar J 16: 376. 28923063 10.1186/s12936-017-2010-x PMC5604301

[62] Runge M , 2020. Simulating the council-specific impact of anti-malaria interventions: a tool to support malaria strategic planning in Tanzania. PLoS One 15: e0228469. 32074112 10.1371/journal.pone.0228469 PMC7029840

[63] Slater HC Walker PGT Bousema T Okell LC Ghani AC, 2014. The potential impact of adding ivermectin to a mass treatment intervention to reduce malaria transmission: a modelling study. J Infect Dis 210: 1972–1980. 24951826 10.1093/infdis/jiu351

[64] Robinson LJ , 2015. Strategies for understanding and reducing the *Plasmodium vivax* and *Plasmodium ovale* hypnozoite reservoir in Papua New Guinean children: a randomised placebo-controlled trial and mathematical model. PLoS Med 12: e1001891. 26505753 10.1371/journal.pmed.1001891 PMC4624431

[65] Ho CI, 1965. Studies on malaria in new China. Chin Med J 84: 491–497. 5865019

[66] Hsiang MS , 2013. Mass drug administration for the control and elimination of *Plasmodium vivax* malaria: an ecological study from Jiangsu province, China. Malar J 12: 383. 24175930 10.1186/1475-2875-12-383 PMC3842644

